# NOVEL TRANSPORTATION SOLUTIONS FOR STRUCTURAL INEQUITIES AFFECTING HOME HEALTH AIDES CARING FOR OLDER ADULTS

**DOI:** 10.1093/geroni/igae098.0874

**Published:** 2024-12-31

**Authors:** Olivia Okereke, Vivian Anable Eme, Cerelia Liu, Farhabi Talukder, Keliane Totten, Joseph Locascio, Anthony Weiner

**Affiliations:** Massachusetts General Hospital, Boston, Massachusetts, United States; Massachusetts General Hospital, Boston, Massachusetts, United States; Massachusetts General Hospital, Boston, Massachusetts, United States; Massachusetts General Hospital, Boston, Massachusetts, United States; Pear Associates, Boston, Massachusetts, United States; Massachusetts General Hospital, Boston, Massachusetts, United States; Massachusetts General Hospital, Boston, Massachusetts, United States

## Abstract

Home health aides (HHAs) provide support for homebound older adults with cognitive impairments, while lessening strain on familial caregivers. However, HHAs face structural challenges in work-related transportation. To address this gap, we began a pilot study of on-demand access to free Uber rides for HHAs who were recruited, trained and provided job placement in partnership with Community stakeholder-partner CCHERS (Center for Community Health Education Research Services). Through community engagement with CCHERS, and as informed by other community partners (e.g., Mothers for Justice & Equality, Mass HomeCare Alliance), we identified lack of affordable, accessible, reliable transportation as the most-cited structural barrier affecting HHAs and their delivery of homecare. Thus, this study provided access to free Uber rides for HHAs’ job training, home care assignments and other work-related travel, for the duration of study funding; all HHAs were offered access to free rides for as long as funding was available, and key metrics were measured among HHAs during periods with and without ride funding. Preliminary data show high prevalence of other social determinants of health among HHAs using rides (e.g., 50% reporting food and housing insecurity). Outcomes among HHAs (work satisfaction, work hours, visit completion) and patients (patients reached, patient diversity) will be presented regarding whether free, on-demand transportation support for HHAs via Uber will: 1) Improve metrics among HHAs (total visits, hours/week and days/week worked, missed visit/no-shows, work satisfaction); 2) Increase racial, ethnic, socioeconomic and geographic diversity of older adults with cognitive impairment/dementia receiving homecare.

